# Concomitant Subtypes of Granuloma Annulare in a 66-Year-Old Female: A Case Report

**DOI:** 10.7759/cureus.46026

**Published:** 2023-09-26

**Authors:** Oscar V Navea, Maria B Navea, Raul De la Fuente

**Affiliations:** 1 General Practice, Universidad de los Andes, Santiago, CHL; 2 General Practice, Universidad de Chile, Santiago, CHL; 3 Dermatology, Hospital Clínico de la Universidad de Chile, Santiago, CHL

**Keywords:** annular granuloma annulare, macular granuloma annulare, patch-type granuloma annulare, granuloma annulare, general dermatology

## Abstract

Granuloma annulare (GA) is a benign, self-limited inflammatory skin condition with an unknown etiology. Although it usually presents with characteristic clinical features, a biopsy may be necessary in atypical cases to differentiate it from other granulomatous diseases. We describe a case of a 66-year-old female with two concomitant subtypes of GA, presenting with distinct clinical features but exhibiting similar histopathological findings. The patient had extensive, pruritic erythematous-violaceous lesions on her lower abdomen, buttocks, and proximal thighs, which had been progressing over the course of one year. Biopsies from the abdominal and thigh lesions showed typical histopathological features of GA, with mucin deposition, histiocytic infiltrate, and granulomatous formations. Treatment with oral antihistamines and medium-potency topical corticosteroids effectively controlled the itching but did not alter the lesion’s appearance. Five months later, the patient developed new, pruritic, skin-colored, confluent papules on the internal face of her left arm, and a subsequent biopsy confirmed annular GA. Although the patient did not follow the prescribed dapsone treatment, the lesions spontaneously regressed within a year. This case emphasizes the importance of recognizing less common presentations of GA, which can mimic other, more concerning conditions. While various therapeutic options have been explored, none guarantee complete remission; however, GA typically resolves on its own over time. A better understanding of the disease's pathogenesis and the development of targeted treatments are warranted to improve management strategies for GA.

## Introduction

Granuloma annulare (GA) is a benign, usually self-limited condition with an unclear pathogenesis [1]. It is characterized by skin-colored or erythematous annular dermal papules and plaques [2,3], which can be asymptomatic or pruritic. The incidence of GA is approximately 0.04%, with the highest frequency observed during the fifth decade of life, being three times more common in women than in men [2].

GA has several clinical variants, including localized, generalized, subcutaneous, perforating, linear, and patch (or macular) types [1]. The papular type is the most common (75%) while the patch type is less frequent [4]. Despite its subtype, GA typically exhibits a characteristic histologic triad of collagen degradation, histiocytic infiltrate, and the presence of mucin [2].

Differential diagnoses will depend on the GA subtype. Although clinical diagnosis is quite accurate, in some occasions, a biopsy will be required to differentiate the lesion from other granulomatous, less benign conditions [2,5]. For instance, patch-type GA can be clinically confused with morphea or mycosis fungoides.

The management of GA will depend on the subtype, lesion location, and characteristics of the patient [5]. Topical and/or intralesional corticosteroids are the first-line treatment for most cases. Other therapeutic options supported by studies include phototherapy and oral immunomodulators like dapsone, hydroxychloroquine, and methotrexate. Although none of these options guarantees complete lesion remission, GA is a self-limited phenomenon and spontaneous regression often occurs even without medical treatment [2].

Here, we present a case of a patient with two concomitant GA subtypes, exhibiting different clinical features but similar histology.

## Case presentation

**Figure 1:**
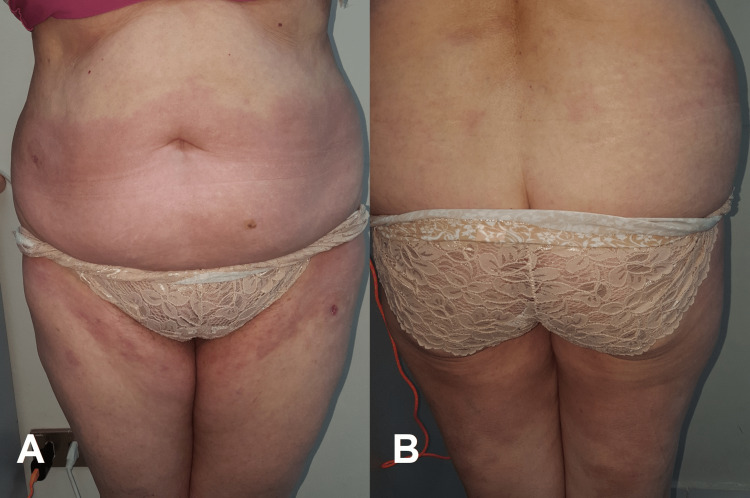
Extensive erythematous-violaceous lesion on the lower half of the abdomen (A), buttocks, and proximal thighs (B).

A healthy, 66-year-old Latin female with no previous medical history or use of chronic or situational medication presented a one-year history of an extensive erythematous-violaceous lesion on the lower half of the abdomen (Figure 1A), buttocks, and proximal thighs (Figure 1B). The lesion had been evolving for a year with increasing intensity of color change and pruritus.

**Figure 2:**
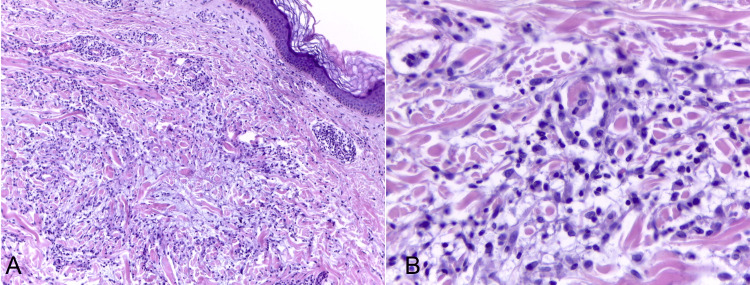
Histopathological slides of abdomen biopsy (A) HE 100x. Skin with orthokeratosis, isolated exocytosis of small lymphocytes, and superficial dermal infiltrate of lymphocytes, mainly affecting the reticular dermis. The infiltrate is organized in poorly defined collections in relation to degenerative collagen. (B) Alcian blue stain 200x. Dermal infiltrate of lymphocytes, histiocytes, and plasmocytes in poorly defined collections in relation to degenerative collagen and increased interstitial mucin.

Two punch biopsies were performed on the abdominal and thigh areas (Figure 2). The histological report described skin with orthokeratosis, isolates exocytosis of small lymphocytes, and superficial dermal infiltrate of lymphocytes, histiocytes, and plasmocytes, mainly affecting the reticular dermis (Figure 2A). The infiltrate was organized in poorly defined collections in relation to degenerative collagen and increased interstitial mucin (Figure 2B), consistent with granuloma annulare.

The patient was started on a second-generation oral antihistamine and medium-potency topical corticosteroids, which effectively controlled the itching but did not alter the lesion's appearance.

**Figure 3:**
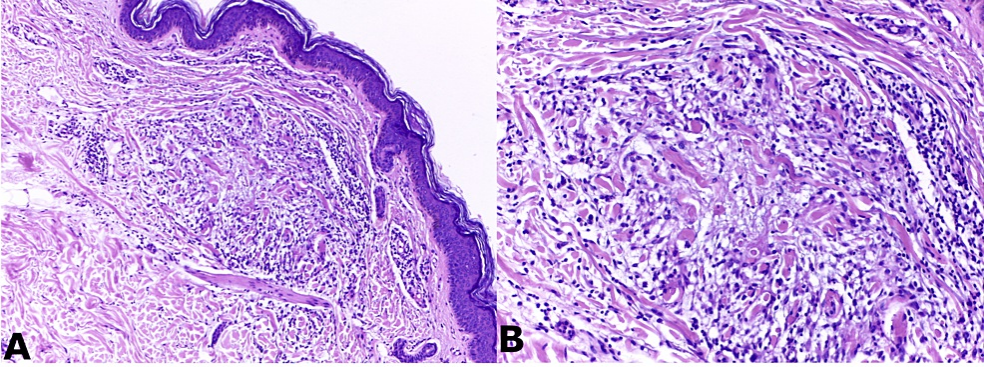
Histological slides from the arm biopsy A) HE 100x. Conserved epidermis, with mononuclear infiltrate in the superficial and middle reticular dermis, in relation to degenerative collagen fibers, confirming a granulomatous structure. B) Alcian blue stain 200x. Mucin presence and a mononuclear infiltrate, mainly histocytes, surrounding degenerative collagen.

Five months later, the patient returned with new pruritic, skin-colored, confluent papules on the internal face of her left arm. A new biopsy confirmed the diagnosis of annular granuloma annulare (Figure 3). Although the patient did not follow the prescribed dapsone treatment, the lesions completely and spontaneously regressed within a year.

## Discussion

Granuloma annulare (GA) is an inflammatory granulomatous skin disease, which can manifest in various forms. In most cases, the clinical diagnosis of GA is straightforward; however, in some instances, a biopsy will be needed to differentiate it from other, less benign diseases.

The differential diagnoses of GA will depend on its presentation although its histology is usually similar regardless of its clinical manifestation. In macular GA specifically, the main differential diagnoses are early morphea and Mycosis fungoides, which can be clinically similar. In such instances, the histological hallmarks of GA include mucin deposits, granulomatous formations with collagen necrobiosis, histiocytic infiltrate, and giant multinucleated cells [2,3].

Regarding the frequency of GA being presented simultaneously as patch-type and papular in different body areas, there have been three reported cases according to Khanna’s review done in 2020, one case report from 2010, and two found in her own 23 patients study [6,7]. Therefore, this case presented consists of a very rare manifestation of GA.

As mentioned above, GA treatment will vary depending on its presentation, considering both the patient’s comorbidities and the affected area. Though not always effective, corticosteroids still are considered the first line of treatment, be it intralesional, topical, or oral [2]. In the presented case, topic corticosteroids were chosen over oral considering both the macule’s size and less minor adverse effects compared to a systemic treatment. Unfortunately, it is described that more than half of the cases of GA respond poorly to this treatment, regardless of its administration, and a second-line treatment is usually needed [2]. Some good options for this are oral treatments with dapsone, hydroxychloroquine, or isotretinoin, though still, none assures consistent nor durable effects [2,8,9]. Phototherapy (specifically PUVA (psoralen and UVA) and PDT (photodynamic therapy)), on the other hand, has also been considered an optimal alternative with good results, but due to their monetary costs and higher risk of squamous cell carcinoma in long treatments, it is left as a second-line treatment, as well as UVB/nbUVB/excimer laser [2,8,10]. The new treatment option studies have been inclined in biologic therapies using adalimumab, etanercept, infliximab, and dupilumab with interesting and promising results, though further research is needed, especially when cases of GA caused by some of these molecules have been reported [2,11,12].

In relation to the psychological sphere, studies trying to find relationships between psychiatric disorders and GA are inconclusive due to the inability to fully discard a misclassification bias, therefore, further studies regarding this aspect are still needed [13]. Regarding our patient, after the itching subsided, she did not refer to any psychological discomfort in relation to her GA, enough so that she did not follow the second-line treatment offered.

## Conclusions

In conclusion, granuloma annulare is a benign, cutaneous disease with a variety of clinical subtypes, which share the same characteristic histological findings. Although clinical diagnosis is often sufficient, a high level of suspicion is necessary, especially in cases of less common presentations, such as macular or patch-type GA, which can mimic less benign diseases like parapsoriasis, morphea, or mycosis fungoides. We presented a rare case of granuloma annulare where both patch and papular types are occurring at the same time, in different parts of the patient's body. As mentioned before, to date, this would be the fourth case reported in the literature.

Advances in biologic therapy show promising results, bringing up a better and more consistent treatment for GA, but there is still much work to be done in this area. Since the pathology of GA is still not fully understood, further studies are also needed in order to establish new therapeutic options. On the other hand, studies on the effect of GA on the patient's quality of life seem like an interesting research area. Moreover, it is essential to remember that none of the current treatments guarantee total remission. However, since GA usually follows a self-limited course, lesions tend to spontaneously subside with time.
